# *Allium cepa* Extract and Quercetin Protect Neuronal Cells from Oxidative Stress via PKC-*ε* Inactivation/ERK1/2 Activation

**DOI:** 10.1155/2016/2495624

**Published:** 2016-09-07

**Authors:** Bo Kyung Lee, Yi-Sook Jung

**Affiliations:** ^1^College of Pharmacy, Ajou University, Suwon 443-749, Republic of Korea; ^2^Research Institute of Pharmaceutical Sciences and Technology, Ajou University, Suwon 443-749, Republic of Korea

## Abstract

Oxidative stress plays an important role in the pathophysiology of various neurologic disorders.* Allium cepa* extract (ACE) and their main flavonoid component quercetin (QCT) possess antioxidant activities and protect neurons from oxidative stress. We investigated the underlying molecular mechanisms, particularly those linked to the antioxidant effects of the ACE. Primary cortical neuronal cells derived from mouse embryos were preincubated with ACE or QCT for 30 min and exposed to L-buthionine sulfoximine for 4~24 h. We found that ACE and QCT significantly decreased neuronal death and the ROS increase induced by L-buthionine-S, R-sulfoximine (BSO) in a concentration-dependent manner. Furthermore, ACE and QCT activated extracellular signal-regulated kinase 1/2 (ERK1/2), leading to downregulation of protein kinase C-*ε* (PKC-*ε*) in BSO-stimulated neuronal cells. In addition, ACE and QCT decreased the phosphorylated levels of p38 mitogen-activated protein kinase. Our results provide new insight into the protective mechanism of ACE and QCT against oxidative stress in neuronal cells. The results suggest that the inactivation of PKC-*ε* induced by phosphorylating ERK1/2 is responsible for the neuroprotective effect of ACE and QCT against BSO-induced oxidative stress.

## 1. Introduction

Oxidative stress has been implicated in neuronal cell death in a variety of neurologic disorders [1–3]. Oxidative stress influences the oxidation states of several proteins in neuronal cells involved in intracellular signaling events, including mitogen-activated protein kinases (MAPKs) and protein kinase C (PKC) [4]. Particularly, MAPKs (extracellular signal-regulated kinase 1/2 (ERK1/2), c-Jun NH_2_-terminal kinase (JNK), and p38MAPK) play a pivotal role during oxidative stress of neurons. Glutathione (GSH) is a major antioxidant in the brain [5], and depleting GSH is quite often accompanied by increased levels of reactive oxygen species (ROS) [6]. We demonstrated previously that the GSH depleting agent L-buthionine-S,R-sulfoximine (BSO) causes cell death by activating PKC-*ε* in brain cortical neurons [2]. We have documented the antioxidant effect of an extract of* Allium cepa *(*A. cepa*), which is a bulbous, herbaceous, biennial monocot plant [7, 8]. Furthermore,* A. cepa* extracts have a neuroprotective effect during transient cerebral ischemia [9] and have antioxidant activity and lipid peroxidation inhibiting properties in the brain [10]. However, it is unclear whether* A. cepa* has protective or antioxidant effects against neurotoxic conditions in a primary neuronal cell culture system. Moreover, the molecular and cellular mechanisms underlying the neuroprotective effect of* A. cepa* extracts against oxidative stress have not been elucidated. Accordingly, in this study, we investigated the protective effect of an* A. cepa* methanol extract (ACE) and its major flavonoid component quercetin (QCT) against oxidative stress-induced neuronal cell death. We established whether MAPKs and PKC-*ε* are involved in the mechanism responsible for the neuroprotective effects of ACE and QCT in neuronal cells during oxidative stress.

## 2. Materials and Methods

All experimental procedures were conducted in accordance with the guidelines on the care and use of laboratory animals set by the Animal Care Committee of Ajou University.

### 2.1. Chemicals

ACE was obtained from Konkuk University. BSO and QCT [2-(3,4-dihydroxyphenyl)-3,5,7-trihydroxy-4H-chromen-4-one] were purchased from Sigma (St. Louis, MO, USA). Trolox [6-hydroxy-2,5,7,8-tetramethylchroman-2-carboxylic acid], commonly used antioxidant, was obtained from Tocris (Ballwin, MO, USA). Z-DEVD-fmk (caspase inhibitor), *ε*V1-2-peptide (PKC-*ε* inhibitor), U0126 [1,4-diamino-2,3-dicyano-1,4-bis(2-aminophenylthio) butadiene] (ERK inhibitor), and SB202190 [4-(4-fluorophenyl)-2-(4-hydroxyphenyl)-5-(4-pyridyl)-1H-imidazole] (p38MAPK inhibitor) were purchased from Biomol Research Labs. Inc. (Plymouth Meeting, PA, USA). Other reagents were of the highest grade commercially available.

### 2.2. Mouse Mixed Cortical Neuronal Cell Culture

Mouse neocortices were obtained from fetal mouse brains on embryonic days 14-15 and grown in Eagle's minimum essential medium supplemented with 21 mM glucose, 5% fetal bovine serum, 5% horse serum, and 2 mM glutamine in 5% CO_2_ at 37°C as described previously [11]. At DIV (days* in vitro*) 7, 10 *μ*M cytosine arabinofuranoside was added to the cultures to halt overgrowth of glial cells. The cells were maintained for 13-14 days and then used for experiments.

### 2.3. Preparation of ACE and Treatment

ACE was obtained using a method modified slightly from a protocol described previously [12]. Briefly, after the outer skins or leaves of fresh* A. cepa* were removed, 50 g of an* A. cepa* bulb was homogenized in 70% methanol (100 mL), and the homogenate was filtered through filter paper. The resulting fractions were lyophilized using a vacuum evaporator (N-2N, Eyela, Tokyo). Lyophilized ACE was dissolved in cell culture medium. The cells were pretreated with ACE (1–10 mg/mL) and QCT (1–10 *μ*M) 30 min before and they were not removed from the culture medium during the BSO treatment.

### 2.4. Lactate Dehydrogenase (LDH) Assay

We assayed LDH released into the medium after BSO treatment to measure overall cell injury by spectrophotometric analysis at 340 nm, as described previously [2, 13]. The percentage of LDH was calculated from the maximum LDH release (100%) induced by lysing cells with 1% Triton X-100.

### 2.5. Terminal Deoxynucleotidyl Transferase dUTP Nick End Labeling (TUNEL) Staining

Fragmented DNA was labeled* in situ* using an Apop Taq Plus kit (Millipore, Gaithersburg, MD, USA). Cells were grown on 24-well plates and fixed in 4% paraformaldehyde in PBS. Nucleosome-sized DNA fragments were tailed with digoxigenin nucleotide and reacted with fluorescein-conjugated antidigoxigenin antibody, as reported previously [14]. Percent cell death was calculated by expressing the number of TUNEL-positive cells as a percentage of total cell count.

### 2.6. Intracellular ROS Level

We followed a method reported previously to determine ROS levels [15]. In brief, cells grown on a glass-bottomed dish were loaded with 10 *μ*M 6-carboxy-2′,7′-dichlorodihydrofluorescein diacetate dicarboxymethyl ester (DCF-DA) in HCSS buffer containing 120 NaCl, 5 KCl, 1.6 MgCl_2_, 2.3 CaCl_2_, 15 glucose, 20 Hepes, and 10 NaOH (mM, pH 7.4) for 20 min at 37°C. DCF-DA was purchased from Molecular Probes (Eugene, OR, USA). The experiments were performed at room temperature on A confocal microscope stage and digitized using FLUOVIEW FV300 (Olympus, Tokyo).

### 2.7. Isolation of ERK1/2, p38MAPK, and JNK from Cell Lysates

ERK1/2, p38MAPKs, and JNK were isolated as described previously [16]. Cells were harvested in RIPA buffer (150 NaCl, 20 Tris-HCl, 1% NP-40, 1% Na-deoxycholate, 1 EDTA, and protease inhibitors at pH 7.4 [mM]) and homogenized, and nuclei and cell debris were removed by centrifugation at 10,000 ×g for 15 min at 4°C. The supernatants were collected for immunoblotting. Protein contents were determined using the BCA*™* protein assay (Pierce Rockford, IL, USA). Protein samples were denatured in Laemmli buffer (4% SDS, 20% glycerol, and 120 mM Tris-HCl at pH 6.8), and total ERK1/2, p38MAPKs, and JNK, as well as their phosphorylated forms, were quantified by immunoblotting using polyclonal and monoclonal antibodies against the proteins (Cell Signaling Technology, Danvers, MA, USA).

### 2.8. Subcellular Fractionation to Isolate PKC-*ε* and Immunoblotting

PKC-*ε* was subcellular fractionated as described previously [16]. Briefly, cells were harvested in homogenization buffer (20 Tris-HCl, 2 EDTA, 5 EGTA, 5 DTT, 6 *β*-mercaptoethanol, 1 PMSF, 0.02 leupeptin, and 10 *μ*g/mL aprotinin, pH 7.4 [mM]) and centrifuged at 100,000 ×g for 1 h at 4°C. The supernatant was retained as the cytosolic fraction. Pellets were resuspended in 1% Triton X-100-containing homogenization buffer and centrifuged at 10,000 ×g for 10 min at 4°C. The supernatant is referred to as the membrane fraction. Protein content was determined using the Bradford protein assay (Biorad, Hercules, CA, USA). The samples were resolved on 8% SDS-polyacrylamide gels and transferred to polyvinylidene difluoride membranes (Millipore, Bedford, MA, USA). The blots were incubated in 5% nonfat dry milk for 1 h at room temperature and then incubated overnight at 4°C with a polyclonal antibody against PKC-*ε* (Santa Cruz Biotechnology, Santa Cruz, CA, USA). The blots were rinsed with Tris-buffered saline and incubated with horse-radish peroxidase-conjugated secondary IgG (Cell Signaling Technology) for 1 h. Bound antibody was detected with an enhanced 3D chemiluminescence kit (Intron, Daejeon, Korea), and the bands were analyzed using a LAS1000 (Fuji Photo Film, Tokyo).

### 2.9. Statistical Analysis

Data are expressed as mean ± standard error of at least three separate determinations in each group. Numerical data were compared using Student's *t-*test or one-way ANOVA* post hoc* test for the unpaired observations between the two groups. A *p* value < 0.05 was considered significant.

## 3. Results

### 3.1. Effect of ACE and QCT on BSO-Induced Neuronal Cell Death

**Figure 1:**
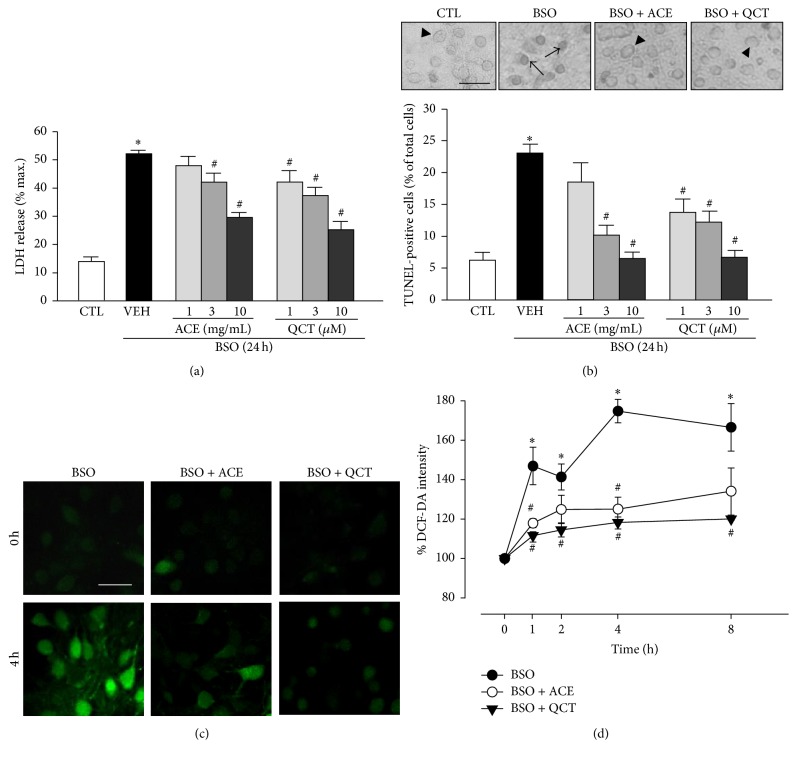
Effects of* Allium cepa* extract (ACE) and quercetin (QCT) on L-buthionine sulfoximine- (BSO-) induced neuronal cell death. (a) Cortical cells were pretreated with ACE (1, 3, and 10 mg/mL) and QCT (1, 3, and 10 *μ*M) for 30 min and then exposed to 10 mM BSO for 24 h. Lactate dehydrogenase (LDH) release was measured after a 24 h BSO treatment. (b) Apoptotic cell death was examined by counting the number of terminal deoxynucleotidyl transferase dUTP nick end labeling- (TUNEL-) positive cells. (Top) TUNEL staining photographs; arrows indicate TUNEL-positive cells, and arrowheads indicate intact cells. (Bottom) Cortical cells were pretreated with ACE (1, 3, and 10 mg/mL) and QCT (1, 3, and 10 *μ*M) for 30 min and then exposed to 10 mM BSO for 24 h. The number of TUNEL-positive (%) cells was calculated by dividing the number of TUNEL-stained cells by the total number of cells after a 24 h BSO treatment. (c) Fluorescence photomicrographs (stained with 10 *μ*M DCF-DA) of cells after a 4 h BSO treatment and the control. Cortical cells were pretreated with ACE (10 mg/mL) and QCT (10 *μ*M) for 30 min and then exposed to 10 mM BSO for 4 h before measuring fluorescence. (d) Reactive oxygen species (ROS) generation during BSO treatment was quantified by pretreating the cells with ACE (10 mg/mL) and QCT (10 *μ*M) simultaneously with 10 mM BSO. ROS levels in the cells were quantified by measuring DCF-DA fluorescence intensity and are represented as the percentage (%) of intensity at 0 min. All data are mean ± standard error (*n* ≥ 5). ^*∗*^ *p* < 0.05 versus control (CTL); ^#^ *p* < 0.05 versus vehicle (VEH).

We measured LDH release after the neuronal cells were treated with BSO to investigate the effects of ACE and QCT on BSO-induced cell death. We pretreated cells for 30 min with ACE (1–10 mg/mL) and QCT (1–10 *μ*M) and then we add 10 mM BSO for 24 h. The concentrations of QCT were selected because the previous studies for neuroprotective effects of QCT suggest a concentration level of maximum 10 *μ*M [17, 18]. In the case of ACE, we previously performed experiments using wide concentration range of the ACE (0.1~1,000 mg/mL) and found no greater efficacy than 10 mg/mL ACE (data not shown) [7]. As shown in Figure 1(a), LDH release increased significantly to 52.1 ± 1.2% in cells exposed to BSO alone for 24 h versus the untreated controls (13.8 ± 1.6%). This increase in LDH release due to BSO was concentration-dependently inhibited by ACE or QCT. A 1 mg/mL ACE concentration did not alter BSO-induced LDH release (47.8 ± 3.3%) but LDH levels decreased slightly (42.0 ± 3.1%) after exposure to 3 mg/mL ACE, and BSO-induced LDH release decreased significantly by 10 mg/mL ACE (29.5 ± 1.7%). Furthermore, QCT had a partially stronger effect on BSO-induced LDH release from neuronal cells than that of ACE. QCT at 1 and 3 *μ*M reduced LDH levels compared with those of the vehicle (to 42.4 ± 4.0 and 37.6 ± 2.9%, resp.), whereas 10 *μ*M QCT significantly reduced BSO-induced LDH release to 25.5 ± 2.9%. To investigate the antiapoptotic effects of ACE and QCT, we analyzed their effects on BSO-induced cell death by TUNEL staining, a well-known indicator of apoptotic cell death [19]. As shown in Figure 1(b), TUNEL positivity of vehicle-treated cells exposed to BSO for 24 h was 23.1 ± 1.5%. When cells were pretreated with different concentrations (1, 3, and 10 mg/mL) of ACE for 30 min and then exposed to BSO for 24 h, the number of TUNEL-positive cells was significantly decreased at 3 and 10 mg/mL concentrations of ACE (to 10.2 ± 1.6 and 6.5 ± 1.0%, resp.), indicating that ACE has an antiapoptotic effect on cortical neuronal cells. Similarly, pretreatment with QCT also significantly reduced the number of TUNEL-positive BSO-treated cells (13.8 ± 2.1, 12.2 ± 1.8 and 6.7 ± 1.1% at 1, 3, and 10 *μ*M, resp.).

### 3.2. Effects of ACE and QCT on BSO-Induced ROS Accumulation

We determined whether ACE or QCT modulated the effect of BSO on ROS. Intracellular ROS levels were measured by fluorescence using DCF-DA. Treating the cortical cell cultures with 10 mM BSO for 8 h increased intracellular ROS levels to a peak at 4 h (174.8 ± 6.0%). Furthermore, a 30 min ACE pretreatment (10 mg/mL) after a 4 h BSO treatment significantly reduced ROS level (125.0 ± 6.1% of control). As observed for ACE, a 30 min QCT pretreatment (10 *μ*M) similarly reduced DCF-DA intensity after 4 h of BSO treatment (118.3 ± 2.8% of control) (Figures 1(c) and 1(d)).

### 3.3. Antioxidant Effects of ACE and QCT Are Mediated by ERK1/2 Phosphorylation and p38MAPK Dephosphorylation

**Figure 2:**
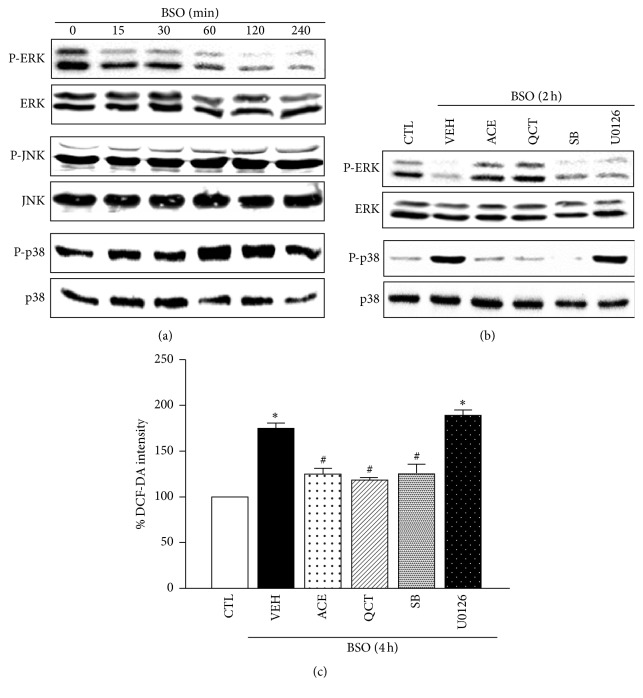
Effects of* Allium cepa* extract (ACE) and quercetin (QCT) on L-buthionine sulfoximine- (BSO-) induced activation of mitogen-activated protein kinase (MAPK) in cortical cells. (a) Representative immunoblots for p-ERK1/2, p-JNK1/2, and p-p38MAPK loading were normalized versus ERK1/2, JNK1/2, and p38MAPK in all neuronal cells exposed to 10 mM BSO for the indicated treatment periods (0–4 h). (b) The cells were treated with 10 mM BSO for 2 h in the presence or absence (VEH) of ACE (10 mg/mL), QCT (10 *μ*M), SB202190 (SB, 10 *μ*M), or U0126 (10 *μ*M). (c) Reactive oxygen species (ROS) generation was quantified during BSO treatment after pretreating the cells with ACE (10 mg/mL), QCT (10 *μ*M), SB (10 *μ*M), or U0126 (10 *μ*M) simultaneously with 10 mM BSO for 4 h. ROS levels in cells were quantified by measuring DCF-DA fluorescence intensity and are represented as a percentage (%) of the control (CTL). All data are mean ± standard error (*n* = 4). ^*∗*^ *p* < 0.05 versus 0 time; ^#^ *p* < 0.05 versus VEH.

We detected MAPK family phosphorylation, such as ERK1/2, JNK1/2, and p38MAPK, in neuronal cells treated with BSO to identify the mediator of the ACE and QCT antioxidant effects against BSO-induced cell death. The ERK1/2 phosphorylation level decreased 60 min after BSO treatment and dropped to its lowest level after 120 min, whereas p38MAPKs increased. In contrast, the JNK phosphorylation level was maintained (Figure 2(a)). BSO decreased ERK1/2 phosphorylation, which was abolished by ACE or QCT, but p38MAPK inhibition by SB202190 and ERK inhibition by U0126 did not recover the downregulation of ERK1/2. In contrast, BSO induced p38MAPK phosphorylation, which was prevented by ACE, QCT, and SB202190, whereas U0126 did not inhibit p38MAPK phosphorylation (Figure 2(b)). Additionally, we investigated the role of ERK1/2 and p38MAPK in neuronal cells during BSO-induced ROS accumulation. BSO-induced ROS accumulation was not inhibited by U0126 (198.2 ± 4.8%), whereas SB202190 significantly inhibited ROS accumulation after 4 h (128.6 ± 10.5%) (Figure 2(c)). These findings suggest that p38MAPK phosphorylation is involved in ROS accumulation, whereas ERK1/2 phosphorylation was not involved in ROS accumulation induced by BSO in neuronal cells.

### 3.4. The Neuroprotective Effects of ACE and QCT Involve ERK Phosphorylation

**Figure 3:**
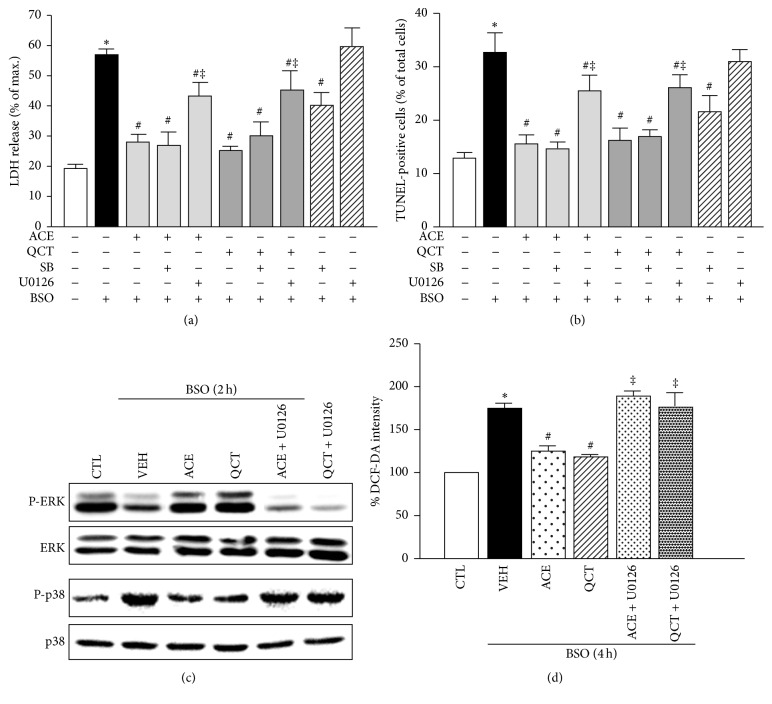
Role of p38MAPK and ERK1/2 phosphorylation in neuronal cells during oxidative stress. (a) Cortical cells were treated with 10 mM L-buthionine sulfoximine (BSO) for 24 h in the presence or absence of the* Allium cepa* extract (ACE) (10 mg/mL), quercetin (QCT) (10 *μ*M), SB (10 *μ*M), or U0126 (10 *μ*M). Lactate dehydrogenase (LDH) release was measured after a 24 h BSO treatment. (b) The number of terminal deoxynucleotidyl transferase dUTP nick end labeling- (TUNEL-) positive (%) cells was calculated by dividing the number of TUNEL-stained cells by the total number of cells after a 24 h BSO treatment. (c) Representative immunoblots for p-ERK1/2 and p-p38MAPK loading were normalized versus ERK1/2 and p38MAPK, respectively. Cells were treated with 10 mM BSO for 2 h in the presence or absence (VEH) of ACE (10 mg/mL), QCT (10 *μ*M), or U0126 (10 *μ*M). (d) Reactive oxygen species (ROS) generation was quantified during BSO treatment after cells were pretreated with ACE (10 mg/mL), QCT (10 *μ*M), or U0126 (10 *μ*M) simultaneously with 10 mM BSO for 4 h. All data are mean ± standard error (*n* = 4). ^*∗*^ *p* < 0.05 versus 0 time; ^#^ *p* < 0.05 versus VEH; ^‡^ *p* < 0.05 versus ACE or QCT.

We conducted LDH release and TUNEL assays to investigate the roles of p38MAPK and ERK1/2 phosphorylation during oxidative stress in neuronal cells. As shown in Figures 3(a) and 3(b), SB202190 blocked the increase in LDH release (40.2 ± 5.3%) and the number of TUNEL-positive (21.4 ± 4.4%) neuronal cells induced by BSO (56.4 ± 2.8 and 33.3 ± 4.3%), but not U0126 (60.9 ± 9.3 and 31.8 ± 2.6%). In addition, we examined whether ERK1/2 phosphorylation regulates the neuroprotective effects induced by ACE and QCT in neuronal cells. As a result, the neuroprotective effects of ACE and QCT against neuronal cell death induced by BSO were partially abolished by U0126 as detected by LDH release (42.7 ± 6.2 and 44.7 ± 9.7%) and the number of TUNEL-positive cells (25.5 ± 3.8 and 26.3 ± 2.6%), respectively, suggesting that ACE and QCT protect BSO-treated neuronal cells via ERK1/2 phosphorylation. Moreover, the inhibition of ERK1/2 by U0126 attenuated ERK1/2 phosphorylation and p38MAPK dephosphorylation induced by ACE and QCT in BSO-induced neuronal cells (Figure 3(c)). As shown in Figure 3(d), we also investigated the effects of ERK1/2 phosphorylation on oxidative stress. U0126 significantly attenuated the antioxidant effects of ACE or QCT at 4 h (150.3 ± 9.5 and 148.3 ± 6.6%), suggesting that the inhibiting effects of ACE and QCT on ROS accumulation are mediated by ERK1/2 phosphorylation in neuronal cells.

### 3.5. The Antioxidant Effects of ACE and QCT Involve Inhibiting PKC-*ε*

**Figure 4:**
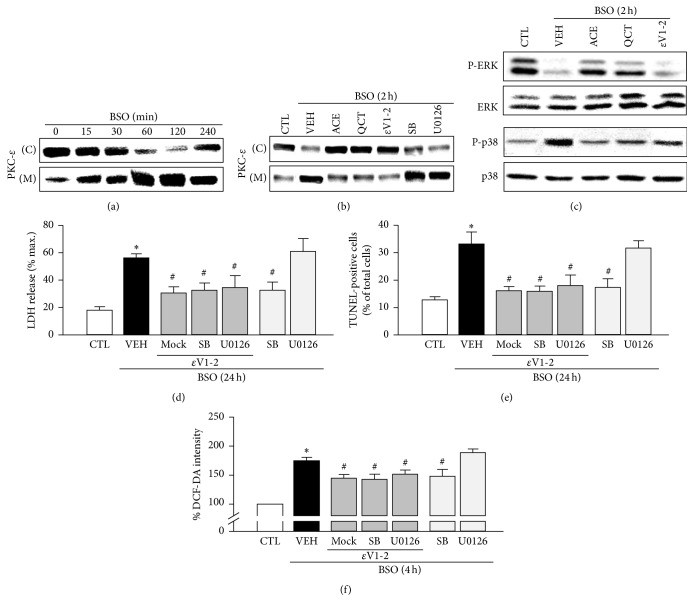
Role of protein kinase C (PKC)-*ε* in the neuroprotective effects of the* Allium cepa* extract (ACE) and quercetin (QCT) against L-buthionine sulfoximine- (BSO-) induced cell death in cortical cells. (a) Western blot analysis of PKC-*ε* in cytosol (C) and membrane fractions (M) of cortical cells exposed to 10 mM BSO for the indicated treatment periods (0–4 h). (b) Representative Western blots of PKC-*ε* in the cytosolic and membrane fractions after treatment with 10 mM BSO for 2 h in the presence or absence of ACE (10 mg/mL), QCT (10 *μ*M), *ε*V1-2 (5 *μ*M), SB (10 *μ*M), and U0126 (10 *μ*M). (c) Representative Western blots of ERK1/2 and p38MAPK after treatment with 10 mM BSO for 2 h in the presence or absence of ACE (10 mg/mL), QCT (10 *μ*M), and *ε*V1-2 (5 *μ*M). (d) Cortical cells were treated with 10 mM BSO for 24 h in the presence or absence of *ε*V1-2 (5 *μ*M), SB (10 *μ*M), or U0126 (10 *μ*M). Lactate dehydrogenase (LDH) release was measured after a 24 h BSO treatment. (e) The number of terminal deoxynucleotidyl transferase dUTP nick end labeling- (TUNEL-) positive (%) cells was calculated by dividing the number of TUNEL-stained cells by the total number of cells after a 24 h BSO treatment. (f) Reactive oxygen species (ROS) generation was quantified during BSO treatment after cells were pretreated with 10 mM BSO for 4 h in the presence or absence of *ε*V1-2 (5 *μ*M), SB (10 *μ*M), or U0126 (10 *μ*M). ROS levels in cells were quantified by measuring DCF-DA fluorescence intensity and are represented as a percentage (%) of the control (CTL). All data are mean ± standard error (*n* = 4). ^*∗*^ *p* < 0.05 versus 0 time; ^#^ *p* < 0.05 versus VEH.

Our previous studies demonstrated that PKC-*ε* is the major PKC isoform involved in pathways triggered by GSH depletion, leading to neuronal death in BSO-treated cortical cells [2]. In the present study, we investigated whether ACE and QCT regulate activation of PKC-*ε* in neuronal cells induced by oxidative stress. After treating the cortical cells with 10 mM BSO, PKC-*ε* levels in membrane fractions increased (~2 h), whereas the PKC-*ε* levels in the cytosolic fractions decreased (Figure 4(a)). ACE or QCT pretreatment strongly inhibited PKC-*ε* translocation in BSO-induced cells (2 h), compared with that by the vehicle. Inhibiting PKC-*ε* translocation with *ε*V1-2 significantly reduced the PKC-*ε* translocation induced by BSO, whereas SB202190 and U0126 did not inhibit the translocation of PKC-*ε* (Figure 4(b)). Additionally, *ε*V1-2 significantly inhibited p38MAPK phosphorylation but did not abolish BSO-induced ERK1/2 downregulation (Figure 4(c)). As shown in Figures 4(d) and 4(e), *ε*V1-2 significantly inhibited LDH release and the number of TUNEL-positive cells after a 24 h BSO treatment (56.4 ± 2.8 and 33.3 ± 4.3 to 30.8 ± 4.3 and 16.2 ± 1.5%, resp.). These effects were similar to those caused by the presence or absence of SB202190 (32.7 ± 5.3 and 16.0 ± 1.9%, resp.), but U0126 did not have an effect (61.0 ± 9.3 and 31.8 ± 2.6%, resp.). Furthermore, *ε*V1-2 partially reduced DCF-DA intensity after a 4 h BSO treatment (174.8 ± 6.0 to 145.2 ± 5.8%) and no additional effects were observed by SB202190 or U0126 (143.1 ± 8.4 or 152.0 ± 6.8%) (Figure 4(f)). These results show that ACE and the major component QCT can reduce neuronal cell death and intracellular ROS accumulation, which is mechanistically linked with PKC-*ε*/p38MAPK signaling.

## 4. Discussion and Conclusion

ACE and its major flavonoid component QCT protect against various neurodegenerative disorders, and their antioxidant activities are believed to prevent neuronal death. Therefore, we focused on the protective and antioxidant effects of the ACE and QCT to identify their molecular mechanisms. Natural dietary antioxidants, such as* A. cepa*, have attracted considerable attention. Both ACE and QCT have broad-ranging pharmacological effects, particularly free radical scavenging properties, that protect against oxidative injury, due to their ability to modulate intracellular signals and promote cell survival [20]. Our results are consistent with previous reports that ACE and QCT can scavenge free radicals, thus, possibly reducing oxidative stress. ACE and QCT has been demonstrated to protect cells from exogenous insults by activating the endogenous defense system, which involves catalase, super oxide dismutase, and glutathione [21, 22]. We sought to identify the signaling system involved in the neuroprotection afforded by ACE and QCT under oxidative stress. Accumulating evidence supports that oxidative stress, including ischemic, inflammation, apoptosis, and other pathological mechanisms, is related to regulation of the MAPK [23] and PKC [24] signal pathways. Our results shed new light on the beneficial effects of* A. cepa* on oxidative stress by demonstrating a potential mechanism by which oxidative stress is caused by neuronal damage. It is widely acknowledged that MAPKs play a critical role regulating neuron responses to oxidative stress [25]. Recent data demonstrate that* A. cepa* or QCT activates MAPK pathways in a variety of cell types, such as endothelial cells [26, 27]. However, neuronal cells have remained unstudied. Phosphorylation of MAPKs is critical for producing oxidative stress in neuronal cells. Therefore, we investigated the effects of ACE and QCT on phosphorylation-mediated activation of MAPKs. MAPKs are comprised of three major subgroups, that is, ERK1/2, JNK1/2, and p38 MAPK, which play key roles transducing various extracellular signals to the nucleus and regulating cell growth, differentiation, and oxidative stress [28, 29]. The dynamic balance between the growth factor-activated ERK1/2 and stress-activated JNK-p38MAPK pathways is important for determining whether neuronal cells survive or die [30]. Indeed, accumulation of phospho-p38MAPK is associated with neurodegenerative diseases induced by oxidative stress, and p38MAPK pharmacological inhibitors have a neuroprotective effect [31]. In contrast, ERK1/2 promotes survival and enhances differentiation of nerve cells [32]. Additionally, ERK1/2 controls direct or indirect antioxidant systems in various cells including neuronal cells [33]. In the present study, we observed that p38MAPK was rapidly activated after BSO exposure, whereas ERK1/2 was downregulated (Figure 2). Our results reveal that ACE and QCT may inhibit p38MAPK phosphorylation by activating ERK1/2, reducing the second increase in ROS, which protected the neuronal cells. Activation of ERK1/2 was noticeable during the antioxidant effect of ACE and QCT and ultimately leads to neuroprotection. This finding suggests a beneficial effect of ERK1/2 against p38MAPK-mediated oxidative stress. Additionally, oxidative stress-induced neuronal cell death mediated by activating MAPK kinases is attributable to modulating the activities of PKC isozymes [34]. PKC-*ε* is an important member of the PKC family that is activated in multiple cell types and is believed to function as both a proapoptotic and an antiapoptotic factor in different mammalian cells [35]. In addition, we demonstrated that BSO induced translocation of PKC-*ε* from the cytosol to the membrane, followed by increased ROS, which led to neuronal cell death [2]. Our results extended the importance of PKC-*ε* in oxidative stress-induced neuronal cell death, and we investigated a possible link between PKC-*ε* and the neuroprotective effects of ACE and QCT during BSO-induced neuronal cell death. Our results show that BSO increased intracellular ROS levels, which were reduced markedly by the PKC-*ε* inhibitor *ε*V1-2 by preventing activation of p38MAPK. One notable observation was that U0126 completely abolished the generation of H_2_O_2_ by ACE and QCT (Figure 3(d)), whereas the ACE and QCT-induced neuroprotective effect disappeared partially after exposure to an ERK1/2 inhibitor (Figures 3(a) and 3(b)). These findings suggest that BSO-induced neurotoxicity may be caused by mediators other than H_2_O_2_ generation and that these other mediators induced by BSO may not be blocked by ACE or QCT. Our results show for the first time that ACE and QCT directly interfere with the activation of PKC-*ε* and p38MAPK and ameliorate the harmful effects of oxidative stress caused by BSO by activating ERK1/2 in neuronal cells. Taken together, our results provide a better understanding of the molecular mechanism of ACE and QCT on protecting neuronal cells and suggest their potential therapeutic effects on various neurodegenerative diseases.
